# Focused Role of an Organic Small-Molecule PBD on Performance of the Bistable Resistive Switching

**DOI:** 10.1186/s11671-015-1148-0

**Published:** 2015-11-16

**Authors:** Lei Li, Yanmei Sun, Chunpeng Ai, Junguo Lu, Dianzhong Wen, Xuduo Bai

**Affiliations:** HLJ Province Key Laboratories of Senior-education for Electronic Engineering, Heilongjiang University, Harbin, 150080 China; School of Chemistry and Materials Science, Heilongjiang University, Harbin, 150080 China

**Keywords:** ITO/PBD/Al, Small molecule, PBD:PMMA nanocomposite film, Nonvolatile bistable resistive switching

## Abstract

An undoped organic small-molecule 2-(4-tert-butylphenyl)-5-(4-biphenylyl)-1,3,4-oxadiazole (PBD) and a kind of nanocomposite blending poly(methyl methacrylate) (PMMA) into PBD are employed to implement bistable resistive switching. For the bistable resistive switching indium tin oxide (ITO)/PBD/Al, its ON/OFF current ratio can touch 6. What is more, the ON/OFF current ratio, approaching to 10^4^, is available due to the storage layer PBD:PMMA with the chemical composition 1:1 in the bistable resistive switching ITO/PBD:PMMA/Al. The capacity, data retention of more than 1 year and endurance performance (>10^4^ cycles) of ITO/PBD:PMMA(1:1)/Al, exhibits better stability and reliability of the samples, which underpins the technique and application of organic nonvolatile memory.

## Background

Organic memory, a multidisciplinary and flourishing frontier of nanotechnology, has succeeded in significant breakthroughs [1–4]. As emerging information medium, devices function as the transmission and manipulation of data, based on organic semiconductor embracing small molecule and polymer. In contrast to inorganic resistive random access memory [5–8], organic resistive random access memory (ORRAM) obtains access to meet the requirements of data storage, large-scale, low-cost, flexible nonvolatile storage for commercialization and utility.

1,3,4-oxadiazole and its derivatives are the group of electron-transport luminescent materials in the domain of organic light emitting diodes (OLEDs). The organic small-molecule material, 2-(4-tert-butylphenyl)-5-(4-biphenylyl)-1,3,4-oxadiazole (PBD), predominantly emits either blue or purple light, offsetting the deficiency of blue or purple luminescent materials [9]. Nonvolatile memory on 1,3,4-oxadiazoles acting as an electron-acceptor mainly focuses on donor-acceptor (D-A) copolymers [10–12]. Transparent material, poly(methyl methacrylate) (PMMA), is used to manufacture various illuminant equipments, optical glass, and optical fiber. For ORRAM, inorganic materials as reported cover quantum dots [13, 14], such as CuInS_2_-ZnS core-shell quantum dots and thiol-capped CdS quantum dots, oxide nanoparticles (NPs) ZnO [15], and carbon nanomaterials like graphene, CNTs, and fullerene together with its derivatives [16–21], which can be embedded into the insulator-like matrix PMMA.

Not only single organic materials, such as poly(N-vinylcarbazole) (PVK) that is widely accepted, but nanocomposites with polymer-polymer or polymer-inorganic blending have unfolded for the research on organic memory. Progressively, ORRAM based on small molecule is attached to great importance. The spin-coated PBD and PBD:PMMA nanocomposite film, first and foremost, were characterized by means of Raman spectrum, scanning electron microscope (SEM), UV–Vis spectroscopy, cyclic voltammetry (CV), and transmission electron microscopy (TEM). The following work highlights the tunable effect of the organic material PBD and its nanocomposite blended by PMMA on electrical properties, and retention and endurance of the resistive switching were additionally detected.

## Methods

2-(4-tert-butylphenyl)-5-(4-biphenylyl)-1,3,4-oxadiazole or PBD:PMMA blends with proportionality of the chemical composition 1:1 was dissolved into the chloroform with the concentration of 0.5 wt.%. At ambient temperature, the solution was stirred by the magnetic stirrer for more than 24 h. Impurities were then removed by the percolation of a 0.45-μm filter. The glass substrate, with the indium tin oxide (ITO, 2000 Å thick) deposited, was sequentially cleaned by the acetone, methanol, and ethanol and proceeded to be kept 40 °C in the vacuum furnace for 30 min. After spin-coating the solution uniformly to fabricate an active layer at 3000 rpm, the solvent was eliminated from the coatings through the vacuum furnace in 70 °C for 2 h. Later, the top aluminum electrode, 300 nm thick, was deposited on the PBD or PBD:PMMA hybrid film and protected by the mask layer with the mask pattern diameter 2 mm. The ITO electrode acts as the anode while the alternative can be seen as the cathode.

## Results and Discussion

**Fig. 1:**
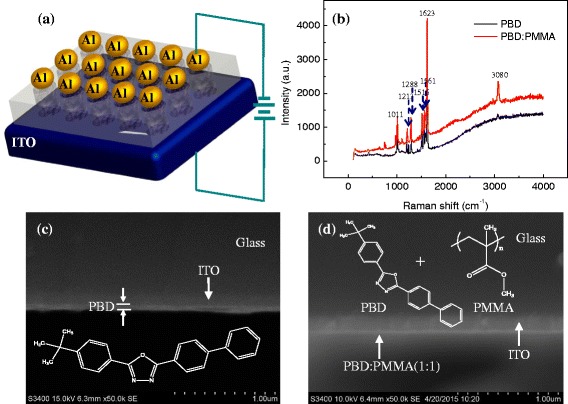
Scheme and characterization of the organic resistive switching based on small-molecule PBD. **a** Sandwiched configuration of ITO/PBD/Al and ITO/PBD:PMMA/Al. **b** Raman spectrum of PBD and PBD:PMMA films. Cross-sectional SEM images for **c** the PBD and **d** PBD:PMMA(1:1) hybrid film. The *insets* in **c** and **d** indicate the organic molecule structures of PBD and PMMA

The sandwiched configuration of both ITO/PBD/Al and ITO/PBD:PMMA/Al is illustrated in Fig. 1a. As shown in Fig. 1b, Horiba Jobin Yvon LabRam HR800 Raman spectroscopy was adopted to test the Raman spectrum of the PBD film and the PBD:PMMA with the ratio of the chemical composite 1:1 coated on the ITO substrate. For the PBD film and PBD:PMMA nanocomposites, the maximum peak is located in 1625 and 1623 cm^−1^, respectively, which is derived from the C–C stretching vibration of the aromatic ring. The rocking vibration peak for the hydrogen atoms on the benzene ring ranges from 500 to 900 cm^−1^, which bears the weak Raman activity. Owing to the C–O–C stretching mode of the 1,3,4-oxadiazole ring, the obvious peak is in 1011 cm^−1^. Ranging between 1514 and 1625 cm^−1^, the stronger Raman activity can be found in that it has the C–C stretching pattern in the benzene ring. Besides, the peak of the C–H asymmetrical stretching vibration in the methyl for the PBD and PBD:PMMA hybrid film is separately 3074 and 3080 cm^−1^, which has the weak Raman activity. Therefore, the stretching culmination, compared with the Raman spectrum of the undoped PBD film, can be boosted by blending PMMA. The cross sections of the undoped PBD nanofilm and doped PBD:PMMA(1:1) nanocomposite film below 50 nm were characterized by xHITACHI S3400-N scanning electron microscope (SEM), exhibited in Fig. 1c, d. Apart from that, NanoMap 500LS Profilometer (aep Technology) was used to measure the thickness of the manufactured PBD as well as PBD:PMMA(1:1) hybrid film. It is shown that a 45.7-nm-thick PBD film was spin coated while the PBD:PMMA(1:1) hybrid film, 37.4 thick, was formed.

**Fig. 2:**
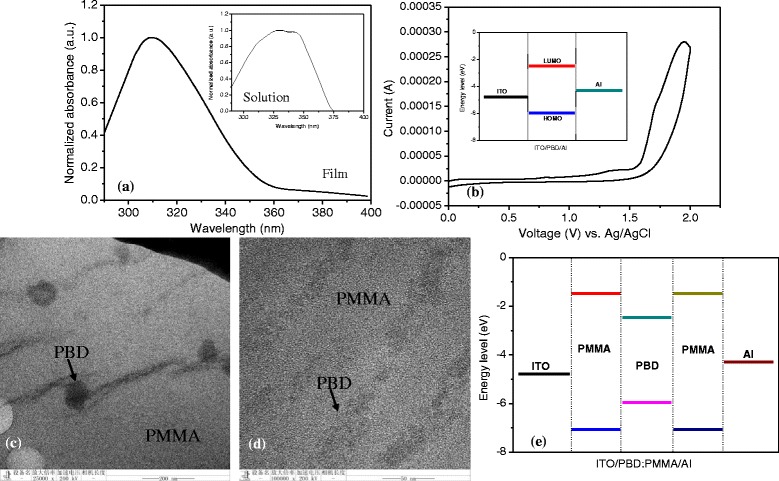
UV–Vis spectra, CV analysis, and TEM for PBD and PBD:PMMA nanocomposite. **a** UV–Vis spectra of the PBD film and solution. **b** CV response of the PBD film. **c**, **d** TEM images of the surface of the PBD:PMMA(1:1) nanocomposite film. **e** Energy band diagram for ITO/PBD:PMMA/Al

UV–Vis spectroscopy and CHI660B electrochemical workstation were utilized to calculate the highest occupied molecular orbital (HOMO) and lowest unoccupied molecular orbital (LUMO) of PBD. The PBD solution and its spin-coated film underwent the optical detection. Observed from UV–Vis absorption spectra in Fig. 2a, the absorption peak *λ*_max_ is located at 309 and 332.5 nm as the absorption edge *λ*_edge_ is in 356 and 374 nm for the PBD film and solution, respectively. Due to *E*_g_ = *hc*/*λ*_edge_, the band gap *E*_g_ of PBD in the form of the film and solution is 3.48 and 3.32 eV, respectively. Cyclic voltammetry (CV) analysis is shown in Fig. 2b, in which the PBD film is measured in the acetonitrile with TBAP (0.1 mol/L). Its onset oxidation potential *E*_ox_ vs. the Ag/AgCl reference electrode is determined to be 1.58 eV. HOMO and LUMO can be expressed as follows:

1 $$ {E}_{\mathrm{HOMO}}=-\left({E}_{\mathrm{ox}}\left(\mathrm{onset}\right)+4.8-{E}_{\mathrm{FOC}}\right) $$

2 $$ {E}_{\mathrm{LUMO}}={E}_{\mathrm{HOMO}}+{E}_{\mathrm{g}} $$

where the reference energy level of ferrocene (FOC) is 4.8 eV, the external standard potential of the ferrocene/ferrocenium ion couple *E*_FOC_ vs. the Ag/AgCl reference electrode is 0.43 eV measured by CV. *E*_HOMO_ (−5.95 eV) and *E*_LOMO_ (−2.47 eV) can be figured out, whose distribution is depicted in inset of Fig. 2b. JEM-2100 transmission electron microscopy (TEM) was adopted to characterize the surface of the PBD:PMMA(1:1) nanocomposite film in Fig. 2c, d, from which the PBD in the PBD:PMMA(1:1) film presents zonal distribution. Thus, the energy band diagram of ITO/PBD:PMMA/Al can be described in Fig. 2e.

**Fig. 3:**
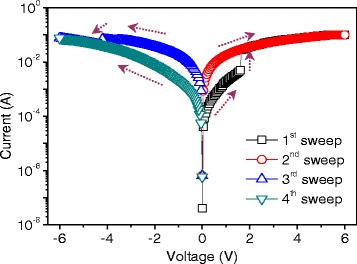
*I*–*V* characteristics of the resistive switching ITO/PBD/Al

2-(4-tert-butylphenyl)-5-(4-biphenylyl)-1,3,4-oxadiazole, as a kind of electron-transport and hole-blocking material, was used to fabricate ORRAM, inserted between ITO and Al electrodes. *I*–*V* characteristics of the resistive switching ITO/PBD/Al were measured by KEITHLEY 4200-SCS semiconductor characterization system, for which the compliance current was restrained to 0.1 A, as indicated in Fig. 3. For starters, the scanning depicts the OFF-state. The steep current change pops up when the device was swept up to *V*_SET_ = 1.6 V. Transferring from turn-off to turn-on, it is denoted as the write process. The following scanning is attributed to the nonvolatile storage that manifests the “read” process. With the resistive switching reverse-biased, the sample shifts from ON-state to OFF-state when the voltage goes down to *V*_*RE*SET_ = −4.2 V. It is the erase process with the negative differential resistive characteristic. All of the experiments above perform the procedure “write-read-erase-read”, which conforms the ITO/PBD/Al has ability in bistable *I*–*V* characteristics, and the threshold voltage is 1.6 V. The current proportion of the low resistive state (LRS) to the high resistive state (HRS) witnesses that it can reach 6. 1,3,4-oxadiazole moiety is regarded as electron-transport group, which can trap and stabilize electrons [10, 12]. The trap/detrap mechanism can be responsible for the bistable *I*–*V* characteristics of ITO/PBD/Al. At positive bias, the injected electrons occupy the charge traps of the PBD film, leading to the current change of ITO/PBD/Al from OFF-state to ON-state when the traps in the organic material are filled with electrons. The electrons are detrapped from the charge-trapping sites and move to the Al electrode under reverse bias, where the rupture of the conductive path contributes to the turn-off of the resistive switching.

**Fig. 4:**
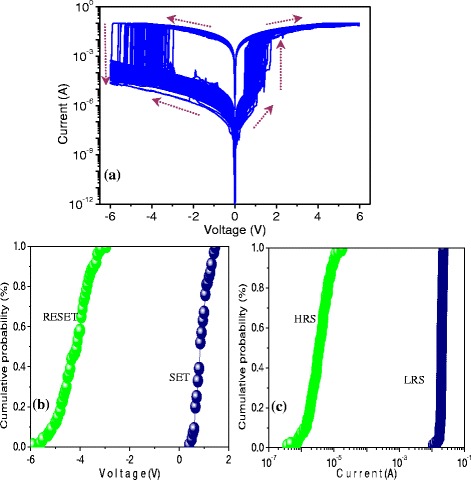
*I*–*V* characteristics of the resistive switching ITO/PBD:PMMA(1:1)/Al. **a** One hundred consecutive cycles of *I*–*V* characteristics of ITO/PBD:PMMA/Al. Data distribution of **b** *V*_SET_ and *V*_RESET_ and **c** *I*_HRS_ and *I*_LRS_ at read voltage −1 V

Polymer nanocomposites are able to exert an exceptional influence on nanoelements in a long-term stability, specifically, to enhance the ON/OFF current ratio of the bistable resistive switching. Thus, PBD:PMMA nanocomposite can be treated as an organic storage material for bistably electrical performance. One hundred consecutive cycles of *I*–*V* characteristics for ITO/PBD:PMMA(1:1)/Al were carried out and plotted in Fig. 4a. The cumulative distribution of *V*_SET_ and *V*_RESET_ is displayed in Fig. 4b, where the mean (standard deviation) of *V*_SET_ and *V*_RESET_ is 0.92 (0.26) V and −4.22 (0.62) V, respectively. At read voltage −1 V, the statistical analysis of ITO/PBD:PMMA(1:1)/Al indicated in Fig. 4c illustrates that the mean (standard deviation) of *I*_HRS_ and *I*_LRS_ is 4.47 (3.31) μA and 19.73 (1.90) mA, respectively. Therefore, the resistive switching ITO/PBD:PMMA(1:1)/Al, as same as ITO/PBD/Al, is electrically bistable. Nevertheless, it has a smaller threshold voltage as well as much larger ON/OFF current ratio close to 10^4^. Insulator-like PMMA has higher *E*_g_ (approximately 5.6 eV) so that carriers require more energy to inject into the e PBD:PMMA nanocomposite film. The current ratio of the resistive switching for PBD blended with PMMA is boosted with the fact that the original state of the resistive switching is in higher resistive state. The mechanism of the resistive switching based on PBD:PMMA nanocomposite can be explained by the trap and detrap of electrons in the PBD material. Electrons are injected from the Al electrode to LUMO of PMMA during forward sweep, by means of tunneling mechanism through PMMA molecules [15], which fill the traps of PBD to fulfill the write process at positive bias. During reverse bias, the electrons captured by the trap of PBD are detrapped and released into the PMMA matrix by Fowler-Nordheim tunneling into the Al electrode. Thus, the resistive switching from turn-on to turn-off originates from the rupture of the conductive path that is the erase process of the storage cell.

**Fig. 5:**
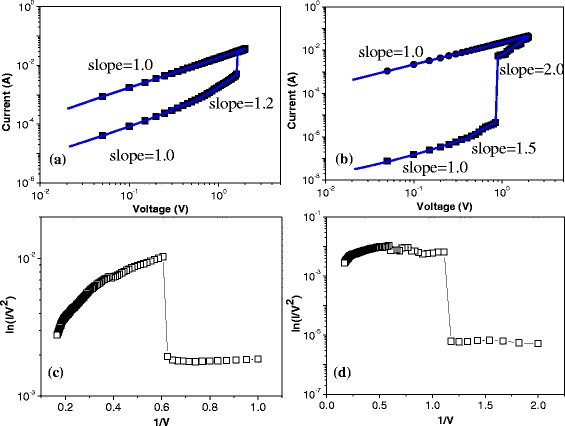
log(*I*)−log(*V*) curves with fitting lines and ln(*I*/*V* ^2^)−1/*V* curves in terms of the conduction mechanism. *I*–*V* characteristics of **a** ITO/PBD/Al and **b** ITO/PBD:PMMA(1:1)/Al in logarithmic profiles. ln(*I*/*V* ^2^)−1/*V* curves for **c** ITO/PBD/Al and **d** ITO/PBD:PMMA(1:1)/Al

With regard to the bistability of the resistive switching for ITO/PBD/Al and ITO/PBD:PMMA(1:1)/Al, the conductive mechanism should be further to analyze. Figure 5a, b illustrates “write-read” process and its fitting curves in the logarithmic coordinate. Below 0.3 V, the slope of ITO/PBD/Al and ITO/PBD:PMMA(1:1)/Al is equally identical to 1.0, in line with Ohmic current conduction. Otherwise, the slope of the fitting lines, before the abrupt current transition, is 1.2 and 1.5, respectively. In particular, the slope of the resistive switching ITO/PBD:PMMA(1:1)/Al can reach 2.0 when the sweep increases from 0.9 to 2.0 V. Therefore, space-charge-limited conduction (SCLC) corresponds to *I*–*V* characteristics as the applied bias is above 0.3 V in the write process. During the read process, the current linearly increases with the incremental voltage, in agreement with Ohmic Law. The results above demonstrate the undoped and doped PBD bistable resistive switches are spontaneously adjusted by SCLC and localized filament. For Fowler-Nordheim tunneling mechanism, the tunneling current as an exponential function of 1/*V* is

3 $$ I\propto {V}^2 \exp \left(-\kappa /V\right) $$

where *κ* is the parameter related with the potential barrier shape. If it is a triangular potential barrier,

4 $$ \kappa =8\pi {\left(2{m}^{\ast}\right)}^{1/2}{\varphi}^{3/2}/3qh $$

where *φ* is the height of the potential barrier, m* is the efficient mass of holes in the polymer semiconductor, and *q* and *h* are the quantity of electric charge and Planck constant, respectively. Depicted in the correlation between ln(*I*/*V*^2^) and 1/*V* in Fig. 5c, d, the write process has the negative resistance conduction because the current dramatically increases during the forward scan, consistent with the Fowler-Nordheim (FN) tunneling model.

**Fig. 6:**
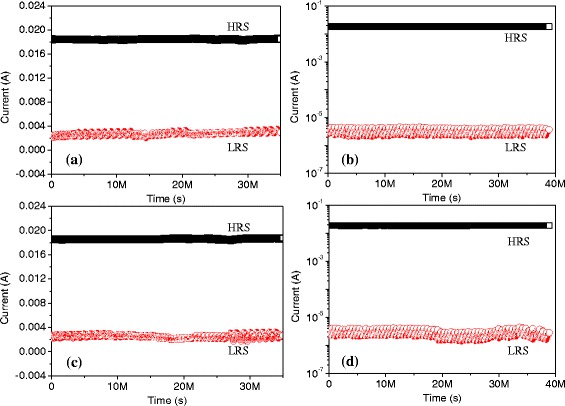
Data retention of the organic resistive switching and linear fit curves. Retention for **a** ITO/PBD/Al and **b** ITO/PBD:PMMA(1:1)/Al at constant bias and their relative retention performance at read cycles with continuous pulses for **c** ITO/PBD/Al and **d** ITO/PBD:PMMA(1:1)/Al

**Fig. 7:**
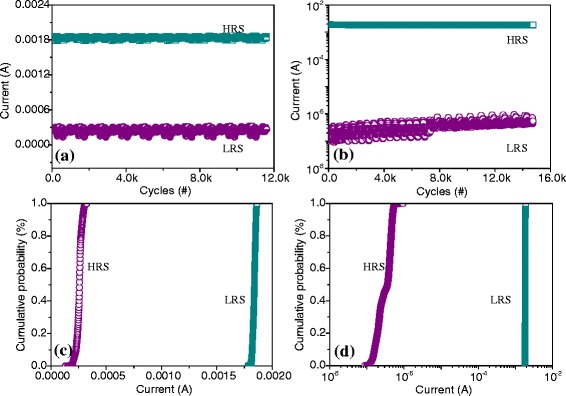
Endurance cycles and statistical analysis of the bistable resistive switching. Endurance cycles for **a** ITO/PBD/Al and **b** ITO/PBD:PMMA(1:1)/Al. Cumulative probability for **c** ITO/PBD/Al and **d** ITO/PBD:PMMA(1:1)/Al

At ambient temperature, the experimental result in Fig. 6 exhibits the ON-state and OFF-state retention of the nonvolatile resistive switching under constant bias *V* = 1 V and read pulses of 1 V with the period 2 μs and the pulse width 1 μs. Figure 6a, c presents the data retention of the bistable resistive switching ITO/PBD/Al at bias *V* = 1 V and read pulses, respectively. In excess of 1-year retention time, the sample, whose ON/OFF current ratio is nearly 6, does not evidently decay. Under the constant voltage and read pulses, the retention of the ITO/PBD:PMMA(1:1)/Al is presented in Fig. 6b, d. With the retention time more than 1 year, this bistable resistive switching in LRS or HRS seems to be more stable and their proportion approaches 10^4^. Aside from that, the bistable switching for ITO/PBD/Al and ITO/PBD:PMMA(1:1)/Al is subject to consecutive endurance cycles (>10^4^), for which Fig. 7a, b exhibits *I*_HRS_ and *I*_LRS_ varying with the cycles at *V*_read_ = 0.1 V. The cumulative distribution of ITO/PBD/Al is displayed in Fig. 7c, where the mean (standard deviation) of *I*_HRS_ and *I*_LRS_ is 0.24 (0.05) mA and 1.84 (0.02) mA, respectively. The statistical analysis of ITO/PBD:PMMA(1:1)/Al indicated in Fig. 7d illustrates that the mean (standard deviation) of *I*_HRS_ and *I*_LRS_ is 0.37 (0.17) μA and 1.83 (0.02) mA, respectively. It is shown that the resistive switching ITO/PBD:PMMA(1:1)/Al has higher ON/OFF current ratio and better read endurance cycles and retention ability, which results from the wide band-gap of PMMA that holds back the carrier transfer. The reason that the initial resistance of the pristine device is far more than that of ITO/PBD/Al leads to the enhanced memory ability, such as ON/OFF current ratio and retention.

## Conclusions

This paper weighs the electrical properties of the undoped and doped PBD films. Although the components of this nanocomposite PBD:PMMA are relatively independent, its performance does not simply add up with the fact that the ON/OFF current ratio is subject to significant enhancement. The bistable behavior of the resistive switching by means of blending PMMA obviously heightens that of the PBD film, which has higher ON/OFF current ratio, and better retention and endurance performance, with the proportionality of the chemical component PBD:PMMA(1:1). Consequently, it provides a widespread prospect for the application of nonvolatile memories.
